# A new automated tool to quantify nucleoid distribution within mitochondrial networks

**DOI:** 10.1038/s41598-021-01987-9

**Published:** 2021-11-23

**Authors:** Hema Saranya Ilamathi, Mathieu Ouellet, Rasha Sabouny, Justine Desrochers-Goyette, Matthew A. Lines, Gerald Pfeffer, Timothy E. Shutt, Marc Germain

**Affiliations:** 1 Groupe de Recherche en Signalisation Cellulaire, Département de Biologie Médicale, Université du Québec à Trois-Rivières, Trois-Rivières, QC Canada; 2 Centre d’Excellence en Recherche sur les Maladies Orphelines-Fondation Courtois, Université du Québec à Montréal, Montréal, QC Canada; 3 Réseau Intersectoriel de Recherche en Santé de l’Université du Québec (RISUQ), Montréal, Canada; 4 Department of Medical Genetics, Cumming School of Medicine, University of Calgary, Calgary, AB Canada; 5 Department of Clinical Neurosciences, Cumming School of Medicine, University of Calgary, Calgary, Canada; 6 Department of Medical Genetics, Alberta Children’s Hospital Research Institute, Cumming School of Medicine, University of Calgary, Calgary, AB Canada; 7 Department of Biochemistry and Molecular Biology, Cumming School of Medicine, University of Calgary, Calgary, AB Canada; 8 Present Address: Department of Engineering, University of Pennsylvania, Philadelphia, USA

**Keywords:** Mitochondria, Cellular imaging

## Abstract

Mitochondrial DNA (mtDNA) maintenance is essential to sustain a functionally healthy population of mitochondria within cells. Proper mtDNA replication and distribution within mitochondrial networks are essential to maintain mitochondrial homeostasis. However, the fundamental basis of mtDNA segregation and distribution within mitochondrial networks is still unclear. To address these questions, we developed an algorithm, Mitomate tracker to unravel the global distribution of nucleoids within mitochondria. Using this tool, we decipher the semi-regular spacing of nucleoids across mitochondrial networks. Furthermore, we show that mitochondrial fission actively regulates mtDNA distribution by controlling the distribution of nucleoids within mitochondrial networks. Specifically, we found that primary cells bearing disease-associated mutations in the fission proteins DRP1 and MYH14 show altered nucleoid distribution, and acute enrichment of enlarged nucleoids near the nucleus. Further analysis suggests that the altered nucleoid distribution observed in the fission mutants is the result of both changes in network structure and nucleoid density. Thus, our study provides novel insights into the role of mitochondria fission in nucleoid distribution and the understanding of diseases caused by fission defects.

## Introduction

Mitochondria require proteins encoded by both nuclear and mitochondrial DNA (mtDNA) to perform their key roles in cellular metabolism. Maintenance of mtDNA copy number and integrity, as well as mtDNA distribution across mitochondrial networks, is crucial for proper mitochondrial function. mtDNA is packed into nucleoprotein complexes called nucleoids. Nucleoids are dynamic structures that actively move within mitochondrial networks and interact with neighboring nucleoids^1^. However, the mechanisms regulating nucleoid maintenance and distribution within mitochondrial networks are still not fully understood.

mtDNA replication is associated with mitochondrial dynamics, the process of mitochondrial fission and fusion^2^. Mitochondrial fusion is regulated by the GTPases Mitofusins (MFN1 and MFN2; outer membrane) and Optic Atropy-1 (OPA1; inner membrane) and is required for the maintenance of mtDNA copy number and integrity^3–5^. Mitochondrial fission is mediated by dynamin related protein 1 (DRP1). DRP1-dependent fission occurs at endoplasmic reticulum (ER)-mitochondrial contact sites (ERMCS) following the initial constriction of the mitochondrial tubule by actin and myosin^6,7^. mtDNA replication occurs at ERMCS^8^. Mutation of the fission proteins non-muscle myosin II (MYH14) or silencing/genetic ablation of DRP1 causes a decrease in nucleoid number, generally without a change in total mtDNA content^9–12^. In the case of DRP1 deletion, these nucleoids are enlarged and confined to an abnormal modified mitochondrial structure called mito-bulbs^9,10,12,13^. While these results suggest that mitochondrial fission is required for nucleoid segregation, it remains unclear how fission contributes to nucleoid maintenance and the spatial distribution of nucleoids within mitochondrial networks.

A small number of studies have previously measured nucleoid distribution using custom scripts on manually annotated images^14–18^. These studies reported either the overall nucleoid density or the average distance between the two closest nucleoids (nearest neighbor distance; nndist), sometimes calculated without considering the positional constraints imposed by the mitochondrial network^14,15^. While these studies give an overview of inter-nucleoid distances, other descriptors describing the global distribution of nucleoids within networks could provide a more informative description of nucleoid distribution. As such, the pair correlation function (pcf) calculates the probability of finding a nucleoid at any distance from a first one within the mitochondrial network, allowing for a finer mapping of nucleoid distribution compared with nndist (which only provides an average distance between adjacent nucleoids). In addition, automation of this quantification would allow more efficient analysis of nucleoid distribution patterns across a large number of mutants affecting nucleoids, leading to a better understanding of nucleoid biology.

Here, we have developed Mitomate Tracker, an automated tool that evaluates the distribution of nucleoids within mitochondrial networks using both nndist and pcf. Using this tool, we demonstrate that nucleoids are distributed in a semi regular fashion within mitochondrial networks, maintaining a minimal spacing between each other. These features were affected by mutations in MYH14 or DRP1, indicating that mitochondrial fission plays an important role in nucleoid distribution and maintenance.

## Results

### Mitomate tracker, a new tool to analyze the distribution of nucleoid across mitochondrial networks

**Figure 1:**
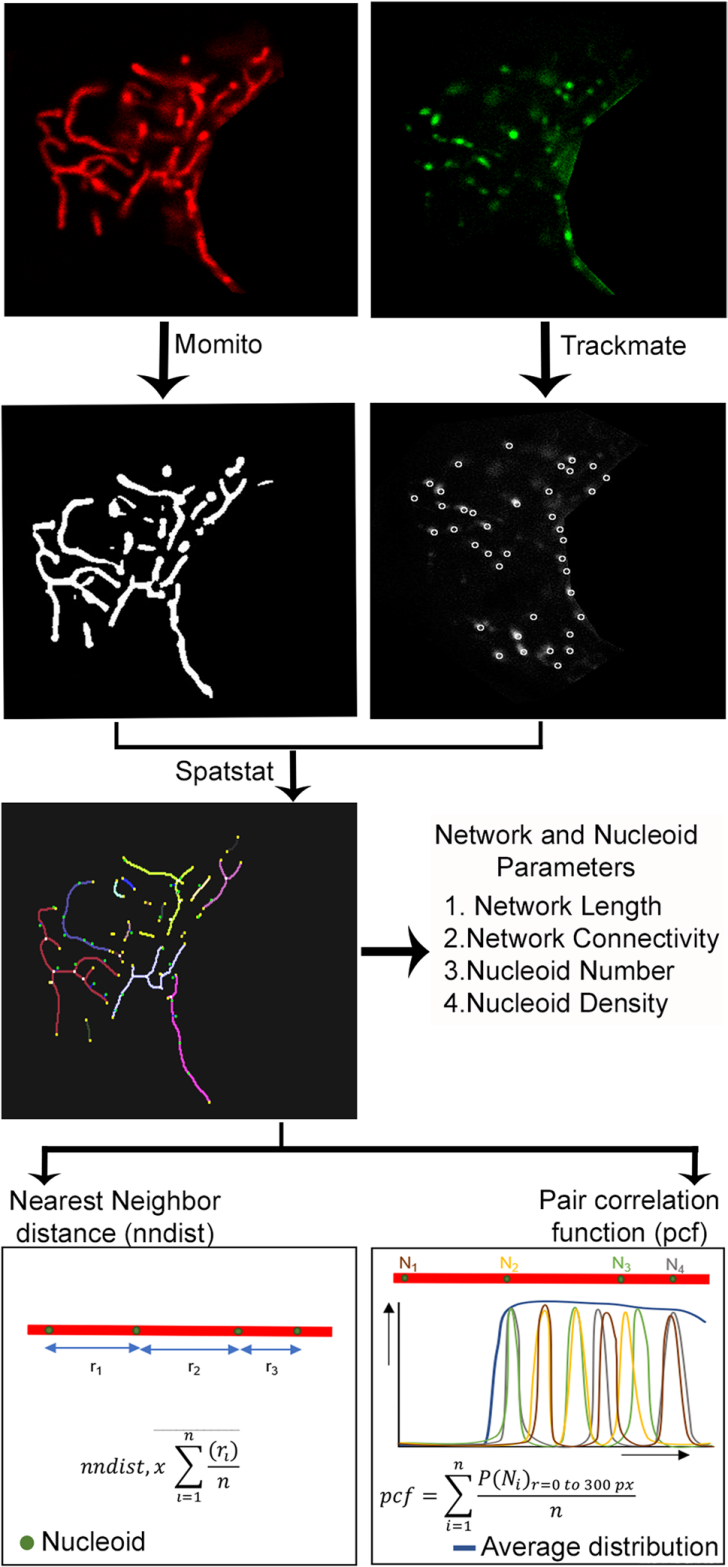
Schematic representation of nucleoid distribution analysis by Mitomate tracker. Confocal live cell Images of mitochondria (TMRM, red) and nucleoids (Picogreen, green—nucleus manually removed) are analyzed using Mitomate tracker. Mitochondria are segmented and their components are identified using Momito while nucleoids are identified using the Image J Plugin Trackmate. The information extracted from Momito and Trackmate is then analyzed by the R package spatstat. Mitomate Tracker provides detailed descriptors of network and nucleoid features and measures nucleoid distribution pattern by two metrics, the nearest neighbor distance (nndist) and the pair correlation function (pcf).

Using the DNA binding dye picogreen, nucleoids can be visualised as punctate structures along TMRM-labeled mitochondria in live cells (Fig. 1). To study nucleoid distribution in an automated manner, we developed an algorithm (Mitomate tracker) that takes advantage of our previously-published mitochondrial network quantification tool (Momito^19^), to which we combined two other tools: the ImageJ plugin, Trackmate which allows nucleoid identification^20^, and the R package, spatstat which calculates point pattern distributions^21^. This results in two distinct outputs: (1) a quantification of network and nucleoid parameters (nucleoid number and density, network length and connectivity), and (2) a point pattern distribution calculated by two distance-based metrics. The first metric, nndist, measures the average distance between a nucleoid and its closest neighbour within the mitochondrial network. The second metric, pcf, estimates the probability of finding a nucleoid at any distance from a first nucleoid within the mitochondrial network (Fig. 1).

### Validating the robustness of nucleoid distribution metrics

**Figure 2:**
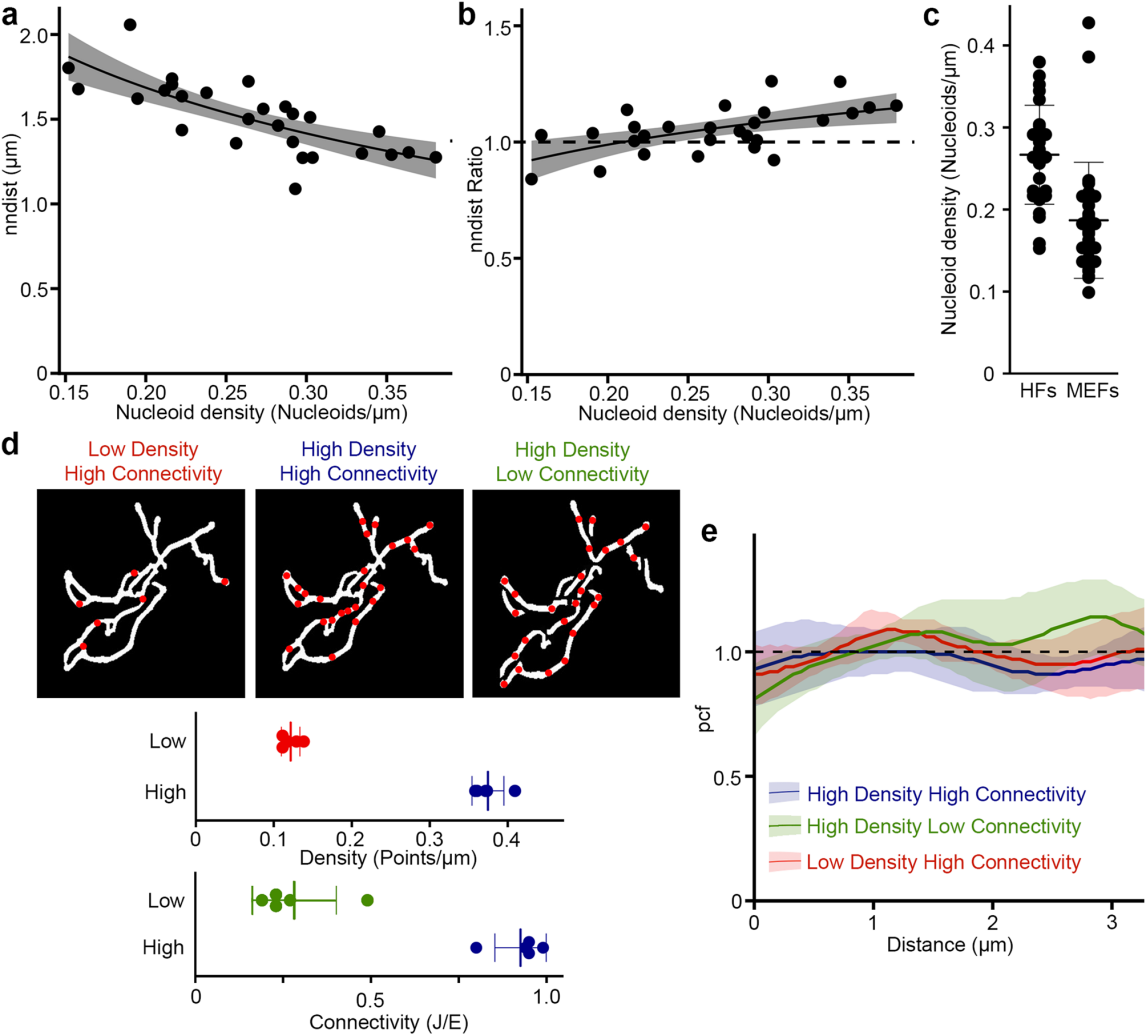
Measurement of nucleoid distribution by Mitomate tracker. (**a**,**b**) nndist is dependent on point density even after normalization. Scatter plot of Actual (**a**) and normalized (relative to an IRP; **b**) nndist values relative to nucleoid density in primary human fibroblasts. Each data point represents an individual cell (n = 27). The dashed line represents a random distribution. Linear regression formula y ~ log(x). (**c**) Nucleoid density in primary human fibroblast (HF) and mouse embryonic fibroblast (MEF) cells. Each point represents an individual cell. (**d**,**e**) Network connectivity and point density do not affect the pcf of random point distribution. (**d**) The networks were either left connected or manually unbranched and overlayed with a low or a high point density. Top, Representative test network used for the analysis. Bottom, measures of point density and network connectivity for the 5 test networks. Each point represents a distinct network. (**e**) pcf curves. Solid lines represent the average point distribution for the indicated conditions and the shaded areas, the SD (n = 5 images). The dashed line represents the expected random distribution.

As previous studies of nucleoid distribution used the average distance between adjacent nucleoids (nndist) as their primary metric^14,16,17^, we first examined the nndist output of Mitomate tracker. Not surprisingly, the absolute distance between nucleoid was dependent on nucleoid density (Fig. 2a; r^2^ = 0.55, p < 0.001). To take this into account, we normalised the actual nndist to an independent random process (IRP), where the same number of points are distributed within the same network independently of each other and network structure. The resulting nndist Ratio should be equal to 1 for a random distribution (actual and random values being equal). However, the normalised nndist was still somewhat dependent on nucleoid density (Fig. 2b; r^2^ = 0.30, p < 0.001). This suggests that nndist is not a robust approach to measure nucleoid distribution owing to its persistent dependence on point density.

We then evaluated the robustness of the pcf. Contrary to the nndist which gives a single average distance per cell, the pcf computes the probability to find a point at any distance of a first point, making it impossible to achieve a simple correlation analysis as used for nndist. We thus used a small number of highly connected mitochondrial networks to which we randomly added points representing the highest and the lowest nucleoid densities found in mouse embryonic fibroblasts (MEFs) and primary human fibroblasts (HFs) (Fig. 2c,d). To avoid measuring effects due to specific random distributions, six different distributions were averaged for each image/condition (see “Methods” for details on the normalisation process). Using these, we then evaluated the influence of point density on the pcf. In this analysis, a random distribution (IRP) has a pcf value of 1 at all distances from the first point (Fig. 2e, black dashed line), values above 1 indicate correlation and values below 1, avoidance (Fig. S1). Consistent with this, points randomly distributed across our test mitochondrial networks resulted in pcf values close to 1 for all densities tested (Fig. 2e; lines, distribution; colored area, SD). Pcf values also remained close to 1 when connectivity was reduced in our test images by manually unbranching the networks within the images using ImageJ (Fig. 2d,e), while keeping point density constant. Overall, our data indicate that the pcf provides a robust approach to study nucleoid distribution within mitochondrial networks.

### Nucleoids have a well-defined organization within mitochondrial networks

To study nucleoid distribution, MEFs and primary human fibroblasts were stained for mitochondria (TMRM) and nucleoids (picogreen) and imaged by confocal microscopy. The images were then processed and analyzed by Mitomate tracker, which identifies individual picogreen foci as a distinct nucleoid (97 ± 1% accuracy of identification, n = 10 cells), irrespective of its mtDNA content (larger nucleoids probably containing more mtDNA copies). This allows us to analyse nucleoid distribution independently of nucleoid segregation following mtDNA replication.

**Figure 3:**
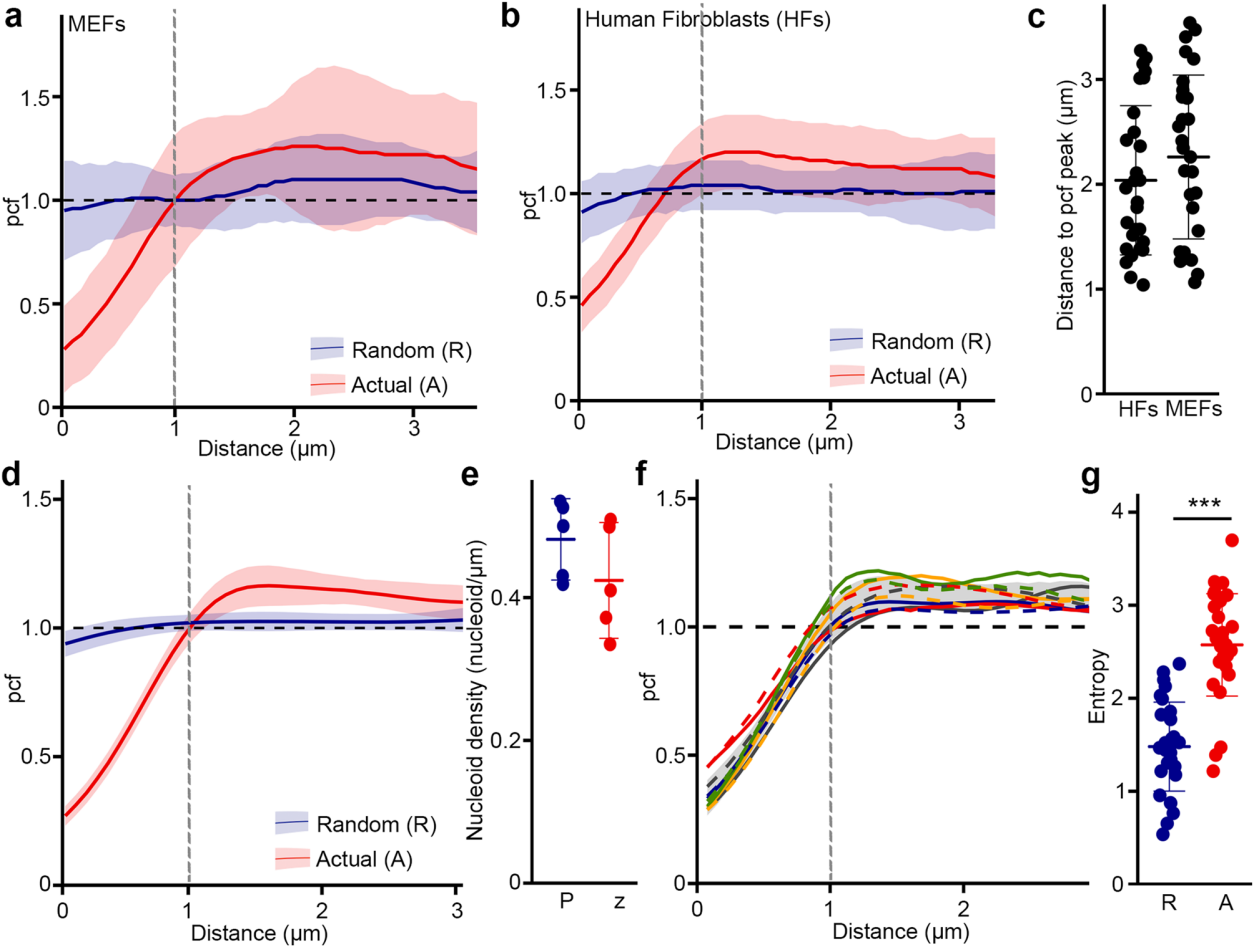
Nucleoid distribution is highly regulated. Pcf curves of nucleoid distribution in MEFs (**a**) and HFs (**b**), Solid lines represent the average of nucleoid distribution and the shaded areas the SD (n = 30 cells). Actual (A, Red) and Random (R, Blue) distributions for the same point densities are shown. (**c**) Distance between nucleoids as determined by the distance of the maximal pcf value. (**d**) Pcf curves of nucleoid distribution in HFs stained with TFAM and TOM20, Solid lines represent the average of nucleoid distribution and the shaded areas the SD (n = 20 cells). The dashed line represents the expected random distribution. Actual (A, Red) and Random (R, Blue) distributions for the same point densities are shown. (**e**,**f**) z-Stacking does not affect pcf results. Control cells were stained for TFAM and TOM20 and the projection of the full z-stack (P) compared to the single focal plane capturing most of the mitochondrial network (z). (**e**) Nucleoid density. Each point represents an individual cell. (**f**) pcf analysis. Each colour represents a distinct cell, the solid line being the single z image and the dashed line the projection. The shaded area represents the average ± SD for the single z images and the black dashed line the expected random distribution. (**g**) Entropy values calculated from MEFs pcf curves in (**a**). Actual nucleoid distribution (A), random distribution (R). Each data point represents one cell. Bars represent the average of 30 cells ± SD. *** p = 2 × 10^–11^. Two-tailed t-test.

The pcf showed a strong nucleoid avoidance at short distances (pcf value < 1) but not at distances greater than 1 µm (pcf value > 1) for both MEFs (Fig. 3a) and primary human fibroblasts (Fig. 3b). The maximal likelihood to find a neighboring nucleoid as estimated from the pcf curve peak for each cell, occurred at 1–3 µm (average 2 µm; Fig. 3c). Similar results were obtained in fixed cells stained for TOM20 (mitochondria) and TFAM (nucleoids) (Fig. 3d). In addition, we tested the effect of cell thickness on the pcf output by comparing a single z-plane with the projection of the entire z stack from the same cell. Consistent with the fact that fibroblasts are flat cells, there were no significant differences between the two sets of images (Fig. 3e,f).

To quantify the differences between the observed nucleoid distribution pattern and an IRP-based distribution, we then measured the entropy (Shannon entropy) of individual pcf curves. In information theory, entropy represents the amount of information present in a variable^22,23^. In the context of a pcf, this means that any horizontal line, including an IRP (pcf = 1) has an entropy of zero, and entropy increases as it deviates from this horizontal line (Fig. S1). The entropy thus provides a measure of the variability of the pcf curve. Accordingly, the entropy values of actual MEFs pcf was significantly higher than for their corresponding IRPs (Fig. 3g). Overall, our results indicate that nucleoid distribution is regulated to maintain a minimal distance between nucleoids, consistent with previous quantifications of inter-nucleoid distances using nndist in yeast cells^16,17^.

### Loss of mitochondrial fission impairs the distribution of nucleoids within mitochondrial networks

**Figure 4:**
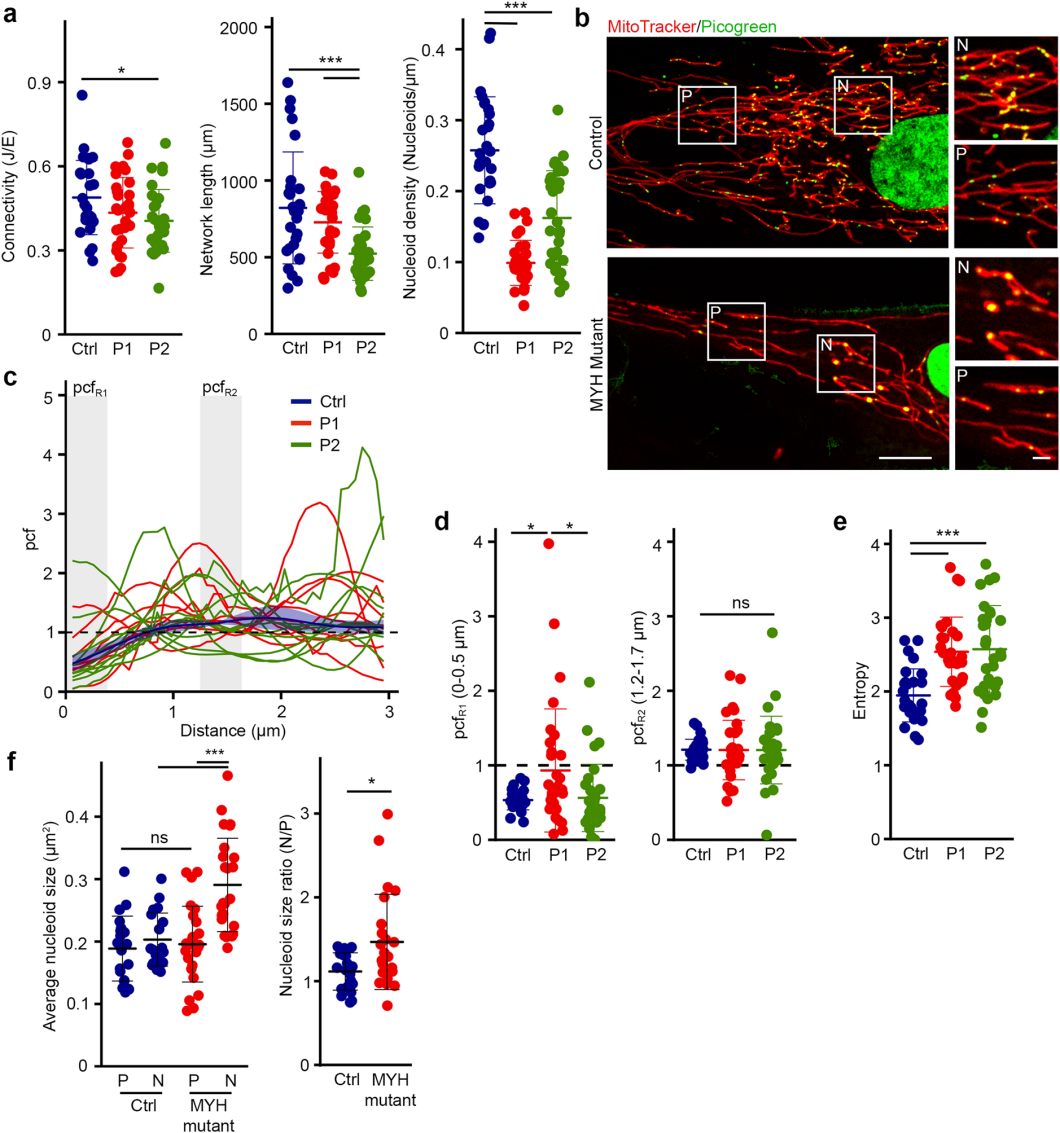
MYH14 is required for proper nucleoid distribution. (**a**) Mitochondrial parameters in Control and MYH14 mutant primary human fibroblasts (P1: Patient 1, P2: Patient 2). Each data point represents one cell. Bars represent the average of 30 cells ± SD. *p = 0.032 (Connectivity, Ctrl vs P2), ***p = 0.0000869 (Network length, Ctrl vs P1), p = 0.0073460 (Network length P1 vs P2), p = 1 × 10^–6^ (Nucleoid density, Ctrl vs P1 / P2). One-way ANOVA. (**b**) Representative live cell confocal images of control and MYH14 mutant primary fibroblasts stained with mitotracker red (mitochondria) and picogreen (DNA) Scale bar, 10 µm; 2 µm for zoomed images. P, periphery; N, perinuclear. (**c–e**) Nucleoid distribution in control and MYH14 primary human fibroblasts. (**c**) pcf curves. The solid line for the control (Blue) represents the average distribution and the shaded area, the SD (n = 8 cells). For the MYH14 mutants, each solid line represents one individual cell (n = 9 cells per patient line), highlighting the variability of the pcf. The dashed line represents the expected random distribution and the grey areas, the distances for which the average pcf was quantified in (d; pcf_R1:_ 0–0.5 µm, pcf_R2:_ 1.2–1.7 µm). (**d**) Average pcf values at short and longer distances. Each data point represents one cell. Bars represent the average of 30 cells ± SD. *p = 0.0182065 (Ctrl vs P1), p = 0.0280752 (P1 vs P2), ns not significant. One-way ANOVA (**e**) Entropy values calculated from the pcf curves in (**c**). Each data point represents one cell. Bars represent the average of 30 cells ± SD. ***p = 0.0000502 (Ctrl vs P1), 0.0000891 (Ctrl vs P2). One-way ANOVA. (**f**) Average nucleoid size in peripheral (P) and perinuclear (N) regions (Left) and nucleoid size ratio (Perinuclear/periphery) of control and MYH14 mutant primary fibroblasts (right). Each data point represents one cell. Bars represent the average of 20 cells ± SD. ***p = 0.0000015 (MYH mutant P vs N), p = 0.0000287 (N Ctrl vs N Mutant), ns not significant, One-way ANOVA (Left). *p = 0.01949, Two-tailed t-test (Right).

Mitochondrial networks are shaped by mitochondrial dynamics, including mitochondrial fusion and fission. Importantly, sites of mitochondrial fission have been associated with sites of mtDNA replication^8^. However, whether this actively contributes to nucleoid distribution within mitochondrial networks and in relation to the nucleus remains unclear. To determine the role of fission in nucleoid distribution, we investigated nucleoid content and distribution in patient fibroblasts with a dominant-negative mutation in MYH14 (R941L), a protein required for the initial constriction of mitochondrial tubules prior to DRP1-dependent scission^10^. MYH14 mutation causes an increase in mitochondrial length^10^ while somewhat decreasing overall mitochondrial content but did not strongly impact mitochondrial connectivity (Fig. 4a). On the other hand, as we previously reported^10^, MYH14 mutant fibroblasts had fewer nucleoids, resulting in a lower overall nucleoid density (Fig. 4a,b).

We then analysed nucleoid distribution in MYH14 mutants. Consistent with MYH14 mutation affecting nucleoid distribution, all mutant cells showed an altered pcf relative to control cells (Fig. 4c, each curve represents a distinct cell). However, we could not observe any conserved pattern across cells, and there were no specific changes in correlation at either short (pcf_R1_, 0–0.5 µm) or longer distances (pcf_R2_, 1.2–1.7 µm) (Fig. 4c,d). In fact, while some cells showed nucleoid clustering at short distances, other cells had a strong avoidance at short distances and a distinct correlation at longer distances (Fig. 4c,d). The increased pcf variability found in MYH14 mutants was also reflected in their increased entropy (Fig. 4e). Overall, the increased variance we observed in the pcf suggests that nucleoids are disorganised in MYH14 mutants in comparison to the control.

We previously reported that MYH14 mutant cells had fewer, but larger nucleoids than control cells^10^. These were evident in mutant cells stained for nucleoids (picogreen) and mitochondria (mitotracker) (Fig. 4b). Importantly, these enlarged nucleoids (possibly cluster of mtDNAs) were restricted to the perinuclear region, which was confirmed by the quantification of nucleoid size close to the nucleus and in the periphery (average size and size ratio; Fig. 4b,f). In contrast, control nucleoids were of similar size irrespective of their localisation (Fig. 4b,f). Thus, our results indicate that MYH14 significantly influences nucleoid maintenance and distribution, supporting the idea that mitochondrial fission is essential for the distribution of nucleoids.

### Dominant-negative mutation in DRP1 causes perinuclear nucleoid clustering and altered nucleoid distribution

**Figure 5:**
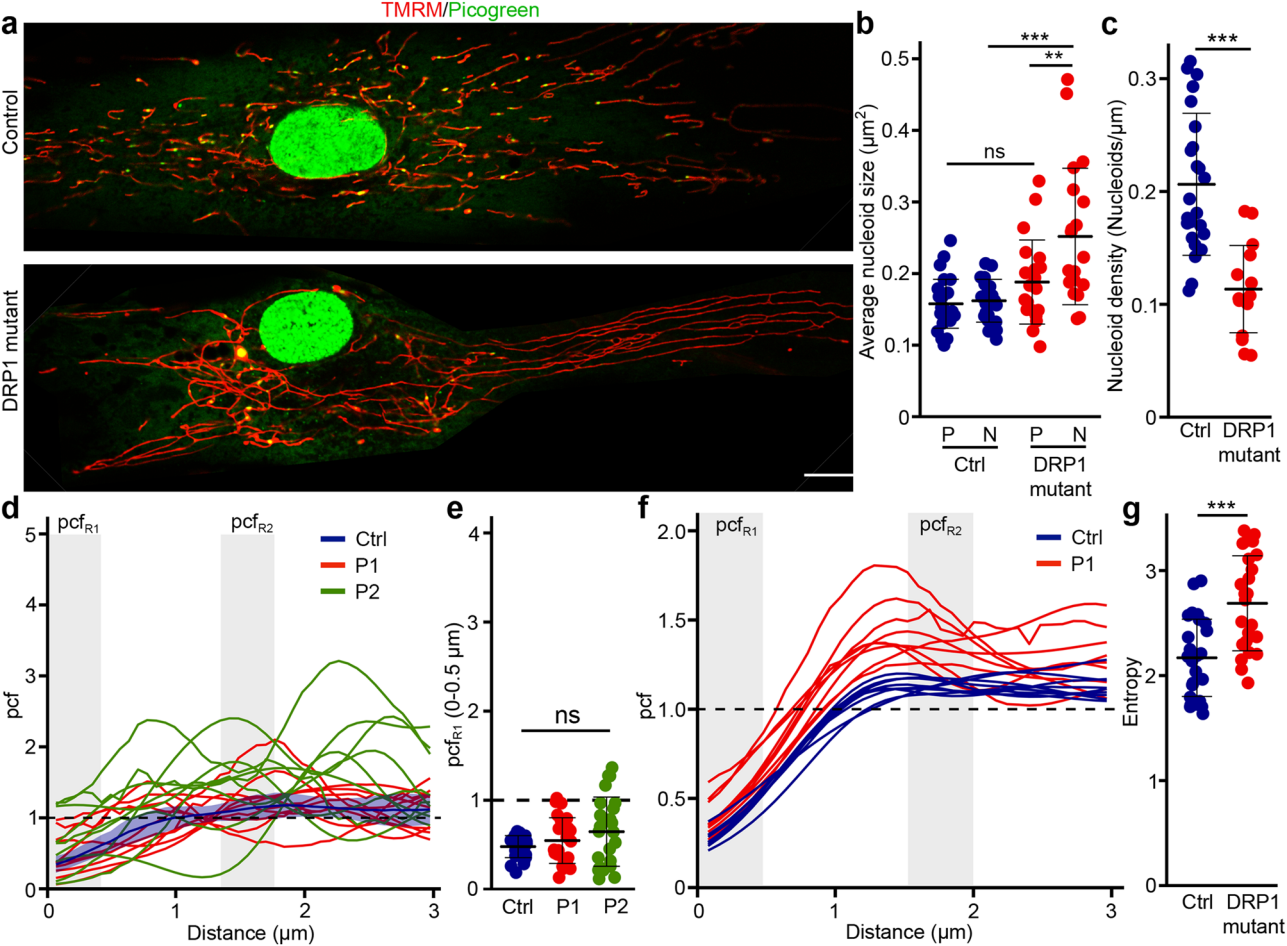
DRP1 is required for proper nucleoid distribution. (**a**) Representative live cell confocal images of control and DRP1 mutant primary fibroblasts stained with TMRM (mitochondria) and picogreen (DNA) Scale bar, 10 µm. (**b**) Average nucleoid size in peripheral (P) and perinuclear (N) regions of control and DRP1 mutant primary fibroblasts. Each data point represents one cell. Bars represent the average of 20 cells ± SD. **, p = 0.0047718 (DRP1 mutant P vs N), ***p = 0.0000130 (N Ctrl vs N Mutant), ns not significant. One-way ANOVA. (**c**) Nucleoid density in control and DRP1 mutant primary fibroblasts. Each data point represents one cell. Bars represent the average of 20 cells ± SD. ***p = 2.164e-06. Two-tailed t-test. (**d**–**f**) Nucleoid distribution in control and DRP1 primary human fibroblasts. (**d**) pcf curves. The solid line for the control (Blue) represents the average distribution and the shaded area, the SD (n = 9 cells). For the DRP1 mutants, each solid line represents one individual cell (n = 9 cells per patient line), highlighting the variability of the pcf. The dashed line represents the expected random distribution and the grey areas, the distance for which the average pcf was quantified (**e**; pcf_R1:_ 0–0.5 µm). (**e**) Average pcf values at short distances. Each data point represents one cell. Bars represent the average of 30 cells ± SD. ns not significant. One-way ANOVA. (**f**) Nucleoid distribution in control and DRP1 primary human fibroblasts stained with TFAM (nucleoids) and TOM20 (mitochondria). Each line represents a distinct cell (n = 9 for control (Blue) and DRP mutants (Red)). The dashed line represents the expected random distribution and the grey areas, pcf_R1_ (0–0.5 µm) and pcf_R2_ (1–1.5 µm)). (**g**) Entropy values calculated from the pcf curves in (**d**). Each data point represents one cell. Bars represent the average of 30 cells ± SD. ***p = 3.377e-05. Two-tailed t-test.

While these results support an important role for mitochondrial fission in the regulation of nucleoid distribution, it remained possible that the nucleoid phenotype we observed in MYH14 mutant cells was restricted to myosin defects. Thus, to confirm the role of fission in nucleoid distribution, we used primary fibroblasts from patients with a mutation in DRP1 (G362D), an essential component of the fission machinery^24^. Mutations in DRP1 or its genetic deletion results in elongated and hyperconnected mitochondria and causes the formation of enlarged nucleoids termed mitobulbs^9,24^. While these previous studies did not determine the subcellular localisation of these mitobulbs, our MYH14 mutant results predict that they accumulate preferentially in the perinuclear region of mutant cells. To verify this, primary human fibroblast cells were stained for mitochondria (TMRM) and nucleoids (picogreen) and imaged by confocal microscopy (Fig. 5a). Similar to MYH14 mutant, the mitobulbs present in DRP1 mutants were mainly restricted to the perinuclear region of the cells (Fig. 5a). In fact, nucleoid size was significantly larger in the perinuclear region of the DRP1 mutants (Fig. 5b). Also, as with MYH14 mutants, the changes in nucleoid size and distribution were accompanied by a reduction in overall nucleoid density (Fig. 5c).

We then measured nucleoid distribution in DRP1 mutants. Similar to MYH14 mutant, the pcf of DRP1 mutant cells was variable, with some mutant cells showing greater correlation at short distances while others avoided each other at short distance (pcf_R1_- 0–0.5 µm) (Fig. 5d,e). Pcf analysis of fixed cells labelled for TOM20 (mitochondria) and TFAM (nucleoids) showed similar pattern (Fig. 5f). As with the MYH mutants, the change in pcf caused by DRP1 mutation also resulted in an increase in entropy (Fig. 5g). Overall, our data indicates that defects in mitochondrial fission impairs proper nucleoid distribution, resulting in their perinuclear clustering and enlargement.

### Synergistic effect of mitochondrial features influences nucleoid distribution in fission mutants

**Figure 6:**
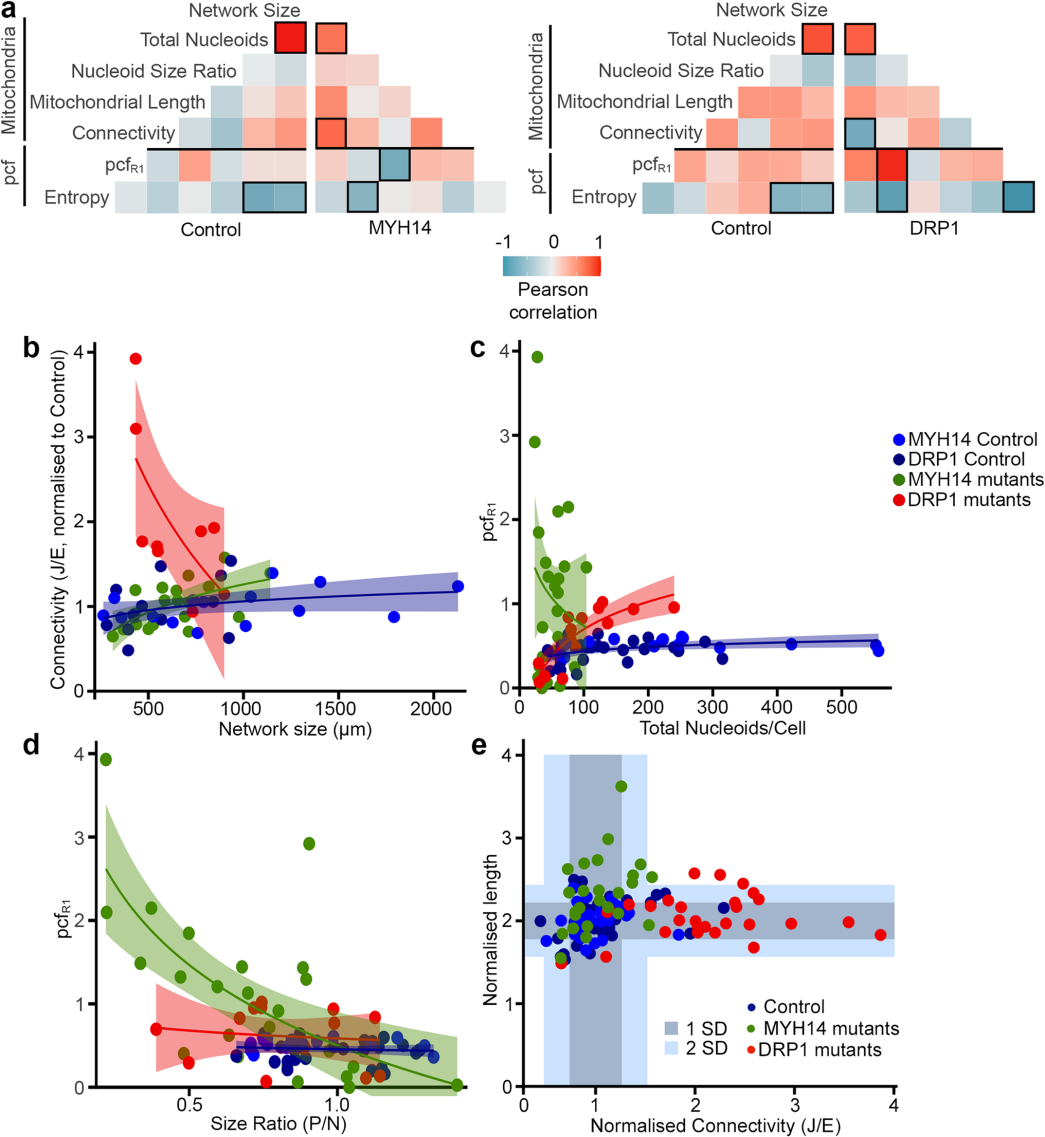
Fission mutants show a distinct relationship between mitochondrial parameters and the pcf. (**a**) Pearson correlation of pcf and mitochondrial parameters for the indicated genotypes. Number of cells used for the analysis: Control MYH14 (24), MYH14 mutants (25), Control DRP1 (17), DRP1 mutants (14) (**b**–**d**) Scatter plots showing the correlation between the indicated parameters for each genotype. The shaded areas represent the 95% confidence interval. Each data point represents one cell (n as in (**a**)). Linear regression formula y ~ log(x). (**e**) Distribution of mitochondrial connectivity and length across genotypes in individual cells. Connectivity was calculated as the number of junctions (J)/number of ends (E) (**e**). For length, we used the number of mitochondria < 2 μm but, to have an increasing value with increasing length, we used 1/this number. All values were normalized to the control for the same experiment. The shaded areas represent 1 SD and 2 SD from the control values. Each data point represents one cell.

To understand how impaired mitochondrial fission leads to such alterations in nucleoid distribution, we first determined whether network features (mitochondrial length, connectivity) and nucleoid parameters (total nucleoids, size ratio) correlated with the pcf changes observed in the two fission mutants. To do this, we calculated Pearson coefficient between each parameter independently for each cell type. Control lines for MYH14 and DRP1 mutants behaved similarly, with the same parameters showing a correlation (Pearson coefficient ≥  ± 0.5, Boxed in Fig. 6a). Among these was a predictable correlation between network size and total nucleoid numbers, but also a correlation between these two parameters and entropy. Importantly, the correlation pattern clearly varied between genotypes (Fig. 6a), suggesting that each genotype behaves differently.

The distinct behavior of each genotype was evident when comparing network features (network size vs connectivity shown in Fig. 6b; Pooled controls r^2^ = 0.07, p = 0.10; MYH14 r^2^ = 0.32, p = 0.006533; DRP1 r^2^ = 0.38, p = 0.04492) but also when nucleoid parameters were correlated with the pcf. For example, the pcf at close distance (Pcf_R1_) correlated with nucleoid number specifically in the DRP1 mutants (Fig. 6a,c; DRP1: r^2^ = 0.77, p = 0.001182; pooled controls: r^2^ = 0.16, p = 0.02054; MYH14: r^2^ = 0.00, p = 0.58), while pcf_R1_ correlated with nucleoid size ratio only in MYH14 mutants (Fig. 6a,d; pooled controls: r^2^ = 0.02, p = 0.53; MYH14: r^2^ = 0.45, p = 0.0001829; DRP1: r^2^ = 0.07, p = 0.67). These results suggest that the differences observed across genotypes are the consequence of distinct changes in mitochondrial features. This is supported by the fact that mitochondrial networks were distinctly affected in MYH14 and DRP1 mutants: MYH14 mutation mainly caused mitochondrial elongation while DRP1 mutants showed a large increase in connectivity (Fig. 6e).

**Figure 7:**
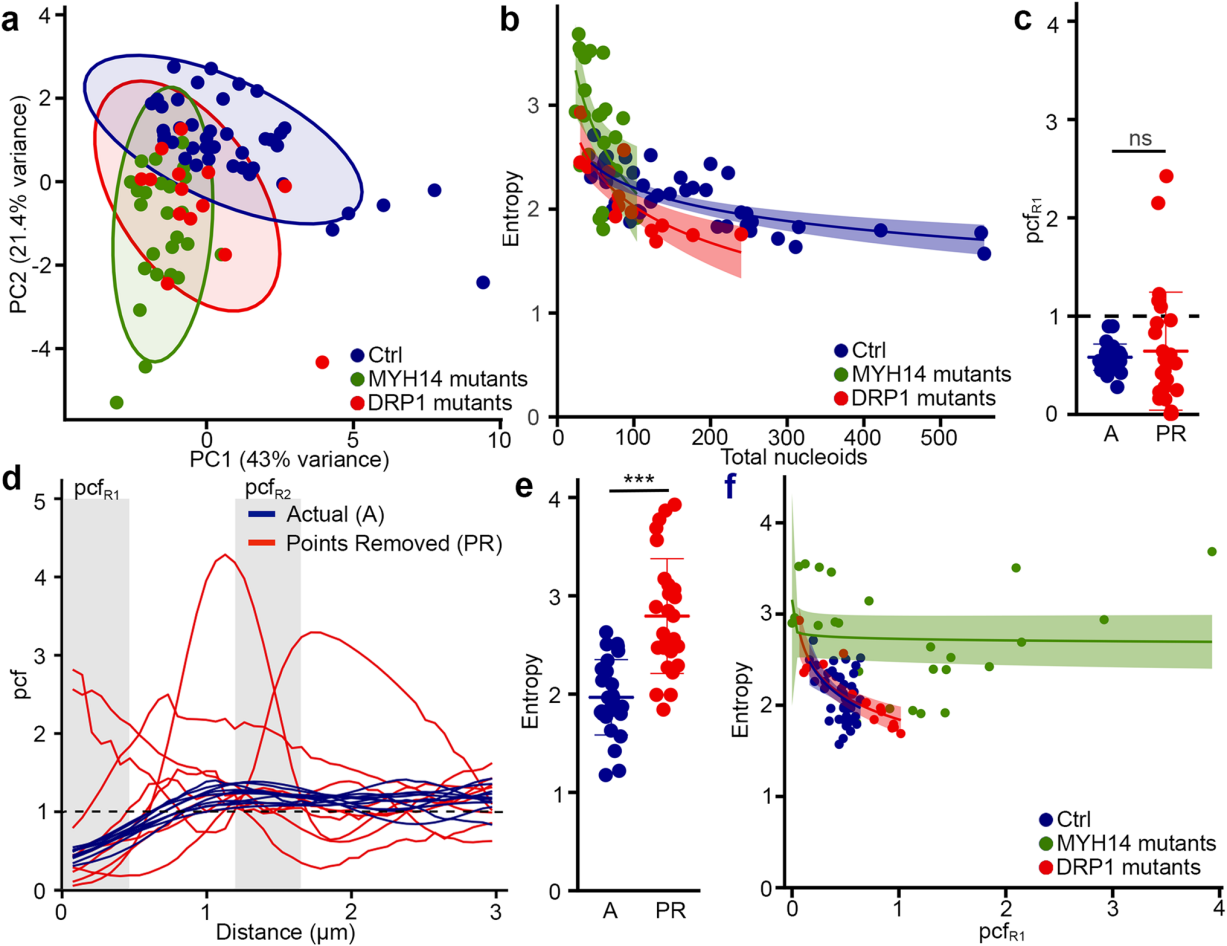
Nucleoid density influences the variability of the pcf curves (Entropy) across genotypes. (**a**) PCA analysis showing the segregation of mutant and control lines. Each data point represents one cell. Circles represent the 95% confidence interval (**b**) Dot plots showing the correlation between Entropy and total number of nucleoids for each genotype. The shaded areas represent the 95% confidence interval. Each data point represents one cell. (**c**–**e**) Decreasing nucleoid density in control cells recapitulates the pcf variability found in fission mutants. Individual cells are shown for pcfR1 values (**c**), pcf curves (**d**) and Entropy (**e**). ns. not significant, p = 1.122e−07, two-sided t-test. (**f**) Relationship between pcfR1 and Entropy in different genotypes. The shaded areas represent the 95% confidence interval. Each data point represents one cell.

On the other hand, a principal component analysis (PCA) of the same genotypes indicated that both mutants segregated away from control cells (Fig. 7a, suggesting that the fission mutants nonetheless share common features relative to mitochondrial network features. In fact, examination of the correlation data indicated that the entropy was correlated with nucleoid content for all genotypes (Fig. 6a; Fig. 7b, overall: r^2^ = 0.49, p = 4.102e−13; pooled controls: r^2^ = 0.49, p = 2.05e−07; DRP1: r^2^ = 0.67, p = 0.0001879; MYH14: r^2^ = 0.26, p = 0.005405), suggesting that pcf variability is a consequence of the smaller number of nucleoids present in the mutant cells (Figs. 4a, 5c). In order to verify whether nucleoid density affects nucleoid distribution, we analyzed nucleoid distribution in control cells where nucleoid density was decreased to match that of MYH14 mutants. To do this, we modified Mitomate tracker to be able to randomly remove points from each input image and analyse it as if it was the actual image (which is distinct from Fig. 2e where several random distributions were averaged—see “Methods”). This resulted in alterations in pcf curves and entropy (Fig. 7c–e) that were similar to those observed in MYH14 mutants (Fig. 4c–e), indicating that nucleoid density influences the nucleoid distribution pattern. Nevertheless, the relationship was different between control cells and MYH14 mutant cells (Fig. 7b, p = 2.2e−16), suggesting that factors other than nucleoid number affect the entropy. This is also supported by the observation that the relationship between the entropy and pcf_R1_ varied across genotypes (Fig. 7f; Pooled controls: r^2^ = 0.17, p = 0.004394; MYH14: r^2^ = 0.02, p = 0.46; DRP1: r^2^ = 0.74, p = 4.482e−05).

Altogether, these results suggest that while both fission mutants globally affect nucleoid features and their distribution in a similar way (Fig. 7a), the manner in which they modulate this process likely differs as a consequence of distinct changes in mitochondrial network features.

## Discussion

Mitochondrial function depends on the proper maintenance of mtDNA and its distribution across the mitochondrial networks. While mitochondrial fusion plays an important role in mtDNA maintenance^25^, the role of mitochondrial fission in this process remains poorly understood. In fact, impaired fission is associated with the presence of enlarged nucleoids but not necessarily a loss of mtDNA content^9,10,12^. In addition, while mitochondrial dynamics have been suggested to control nucleoid distribution across mitochondrial networks, the underlying mechanisms remain poorly understood. To address these questions, we developed an automated tool, Mitomate tracker to quantify nucleoid distribution within mitochondrial networks. Mitomate tracker allows to use minimally pre-processed images to provide detailed quantitative data on mitochondrial network and nucleoids features and distribution. We initially measured both nndist and pcf to quantify nucleoid distribution. However, we found that the pcf is a more robust metric than the nndist. This is likely because the pcf globally measures the distribution probability relative to an IRP instead of the single average distance provided by the nndist. In addition, unlike the nndist, the calculation of the pcf probability takes into account the fact that points are closer when density is higher, providing a normalization relative to density. While we used it here to demonstrate the importance of mitochondrial fission in the regulation of nucleoid distribution, it could be utilized to characterize the distribution of any mitochondrial proteins dispersed as individual foci along mitochondrial networks.

Our results indicate that in healthy cells, nucleoids are distributed in a semi-regular manner, with nucleoids strongly avoiding each other at closer distances (Fig. 3a,b). This is consistent with previous manual nndist measurements in yeast and human cells^15,16^ and strongly support the notion that nucleoid distribution is actively regulated in the cells.

Recently, we have shown that mutation of the mitochondrial fission protein MYH14 reduced total nucleoid population without altering mtDNA content^10^. Similarly, knock down or genetic ablation of DRP1 altered nucleoid content by causing the formation of mitobulbs^9,12,13^. As these results suggest a potential role for fission in nucleoid maintenance, we have used both mutants to directly address the role of mitochondrial fission in the regulation of nucleoid distribution. Our results show that inhibiting mitochondrial fission disrupted nucleoid distribution as reflected by the high variability of the pcf curves and the perinuclear accumulation of enlarged nucleoids in both MYH14 and DRP1 mutants. Specifically, some mutant cells showed strong short distance correlation while in others, nucleoids avoided each other. The reduced nucleoid density observed in MYH14 and DRP1 mutants significantly contributed to this variability (entropy). Nevertheless, the specific contribution of distinct nucleoid or network parameters to the pcf varied across genotypes, suggesting a complex interplay between nucleoid and mitochondria network topologies in regulating nucleoid distribution. Consistent with this, mitochondrial networks were distinctly affected in MYH14 and DRP1 mutants, likely reflecting the distinct role of these proteins in mitochondrial fission. While DRP1 is an essential fission protein required for the physical severing of mitochondrial tubules, MYH14 encodes one of three non-muscle myosin II proteins that are involved in the initial ER-mediated constriction of the mitochondrial tubule^10,26–28^. In addition, we previously found that the mitochondrial phenotype of MYH14 mutants was most evident in the peripheral area of the cells^10^, which could further affect the nucleoid distribution pattern.

The fact that our findings differed between the MYH14 and DRP1 mutant cells is not surprising, given the disparate clinical phenotypes in the patients from whom they were isolated. The MYH14 patients developed axonal sensorimotor neuropathy and sensorineural hearing loss^10^, whereas the DRP1 patient had severe central nervous system involvement^24^. A question of interest for future study with this tool may be to determine whether it can resolve differences in nucleoid distribution correlated to phenotypic variation from mutations in the same gene, implicating differing biological mechanisms for alternate clinical presentations. For example, MFN2 is a major human disease gene associated with numerous mitochondrial functions (including mitochondrial fusion) and highly variable clinical phenotypes, which are not consistently correlated to cellular phenotypes^29^. In the specific case of MYH14, other mutations result in isolated and severe hearing loss or later-onset sensorineural hearing loss^30–32^. In the case of DRP1, phenotypes can vary, and recently severe cardiac involvement has been described from a novel mutation^33^. The automated and quantitative approach described in this work presents an additional tool to assess these differences at the cellular level.

It is nevertheless important to note that in our setup, mtDNA clusters less than about 300 nm apart are not resolved and thus do not contribute to the pcf curves. While this probably does not affect wild-type cells, this could alter the analysis of the fission mutants as they contain enlarged nucleoids that likely represent a cluster of mtDNAs that failed to separate following mtDNA replication. It is thus possible that, under conditions where individual mtDNA molecules could be resolved, the pcf would detect a strong correlation at very short distances (< 300 nm) in these cells. In addition, the thickness of the cells used for imaging could affect the outcome of the pcf. Here, we used flat cells (fibroblasts), which allowed us to consider single plane images without affecting the overall results. However, while Momito has been designed to address the 3D structure of mitochondrial networks^19^, the proper identification of mitochondria and nucleoids could be affected in thicker cells, altering the pcf analysis. Nevertheless, our data supports the idea that mitochondrial fission regulates nucleoid distribution and prevents nucleoid clustering to facilitate homogenous distribution of nucleoids within mitochondrial networks.

In conclusion, our results indicate that, while mitochondrial fission might not directly control mtDNA replication, it plays an essential role by regulating nucleoid distribution across mitochondrial networks. This process is likely required to facilitate homogenous distribution of mtDNA and OXPHOS protein subunits and its alteration in fission mutants likely contribute to the development of associated pathological conditions.

## Methods

### Reagents

Cell culture reagents were obtained from Wisent. Other chemicals were purchased from Sigma-Aldrich, except where indicated.

### Cell culture and live cell imaging

Primary human fibroblasts (controls, MYH14 mutants and DRP1 mutants) were generated from skin biopsies, collected as part of an approved research protocol (University of Calgary Research Ethics Board (MYH14 mutants), Research Ethics Board of the Children’s Hospital of Eastern Ontario (DRP1 mutants)), and written informed consent from participants was obtained. This study was performed in accordance with the Declaration of Helsinki. Biopsy samples were processed as described and cultured in Dulbecco’s modified Eagle’s medium (DMEM) containing 10% fetal bovine serum (FBS), supplemented with Penicillin/Streptomycin (100 IU/ml/100µL/mL)^24,34^. Immortalized Mouse Embryonic Fibroblasts (MEFs) were cultured in DMEM supplemented with 10% fetal bovine serum. For live cell imaging, cells were seeded onto glass bottom dishes and stained for 30 min with 250 nM TMRM (Thermo fisher Scientific, T668) (MEFs and DRP1 mutant and control human fibroblasts) or 50 nM Mitotracker Red (Thermo fisher scientific, M7512) (MYH14 mutant and control fibroblasts) and the DNA dye PicoGreen (Thermo Fisher Scientific, P11495) (3 µL/mL). After staining, cells were washed 3 times with pre-warmed 1× phosphate buffered saline (PBS), and normal growth media was added prior to imaging.

### Microscopy

Images for MEFs, DRP1 mutant fibroblasts and their wild-type control were acquired with a Leica TSC SP8 confocal microscope fitted with a 63×/1.40 oil objective using the optimal resolution for the wavelength (determined using the Leica software). Images from MYH14 cells and their control were taken with an Olympus spinning disc confocal system (Olympus SD-OSR) (UAPON 100XOTIRF/1·49 oil objective) operated by Metamorph software. The SD-OSR was equipped with a cellVivo incubation module to maintain cells at 37ºC and 5% CO_2_ during live cell imaging.

### Image analysis using Mitomate tracker

Red and green channels were separated, nuclei were manually removed from the Picogreen channel, and the images converted to 8-bit using ImageJ. The mitochondrial channel was then segmented using the ImageJ filter tubeness and global thresholding. To measure the correlation between nucleoids on the mitochondrial network, the images were analyzed using the R package spatstat (flowchart in Fig S2). This analysis requires two inputs: a linear network (mitochondria) and a point pattern (nucleoids). Mitochondrial components (tubules, ends (E), junctions (J)) were extracted from the segmented mitochondrial image and the most probably network configuration determined using Momito^19^.While Momito takes into account the presence of overlapping tubules for its analysis of the most probable mitochondrial network, care was taken to use cells that have a flat and clearly identifiable mitochondrial network to avoid issues related to cell thickness. This was used as the input for the linear network. Nucleoids were identified using the ImageJ plugin TrackMate^20^ and the coordinates of the nucleoids associated with the mitochondrial network (within 6 pixels center to center) used to generate the point pattern. Overall 97 ± 1% of the nucleoids were properly identified in control cells, although 38%of the larger nucleoids (> 0.6 µm) present in mutant cells were identified as 2 or more nucleoids. Each nucleoid was assigned to a single mitochondrial cluster (connected mitochondrial tubules) and only nucleoids within the same cluster were analysed together.

The pcf analysis was then carried using the spatstat *linearpcf* function for distances from 0 to 300 pixels (although distances < 5 pixels (< 0.3 µm) are within the resolution limit of the images) with a bias correction at each end of the interval (*correction* = *"Ang"*) and a bandwidth of 5 pixels (corresponding to the size of control nucleoids), while the nndist was calculated using the spatstat *nndist* function. As Spatstat computes the pcf for individual mitochondrial clusters, we had to sum the contribution of each cluster to generate the total pcf by taking into consideration the size of each mitochondrial cluster and the number of nucleoids that it contains. This was achieved as follows:

In Spatstat, the estimator for the pcf ${\widehat{g}}_{k}\left(r\right)$ for a given subgraph ${G}_{k}$(a mitochondrial cluster) is given by

$${\widehat{g}}_{k}\left(r\right)=\frac{l\left({G}_{k}\right)}{{n}_{k}\left({n}_{k}-1\right)}\sum_{i=1}^{{n}_{k}}\sum_{j\ne i}\frac{\kappa \left({d}_{{G}_{k}}\left({x}_{i},{x}_{j}\right)-{r}_{k}\right)}{m\left({x}_{i},{d}_{{G}_{k}}\left({x}_{i},{x}_{j}\right)\right)}$$

where $\kappa$ is the gaussian kernel of 5 pixels used for smoothing and m is analogous to the perimeter for a network of radius ${d}_{{G}_{k}}\left({x}_{i},{x}_{j}\right)$ around the point ${x}_{i}$. The length of the subgraph is $l\left({G}_{k}\right)$ and it contain ${n}_{k}$ points. We have normalized the pcf ${\widehat{g}}_{k}\left(r\right)$ for the whole network (graph, $G$) by

$${\widehat{g}}_{G}\left(r\right)=\frac{\sum_{{G}_{k}\in G}l\left({G}_{k}\right)}{\sum_{{G}_{k}\in G}{n}_{k}\left({n}_{k}-1\right)}\sum_{{G}_{k}\in G}\frac{{n}_{k}\left({n}_{k}-1\right) {\widehat{g}}_{k}\left(r\right) }{l\left({G}_{k}\right)} .$$

In addition, to avoid spurious effects caused by variation in nucleoid density and network features (length and connectivity), we normalised both nndist and pcf by dividing the observed value (actual nndist or pcf) by the value obtained using an IRP with the same point density distributed across the same network. For the pcf, this was done for each distance $\left(r\right)$ measured. The randomised point pattern used to correct for network effects was generated using the spatstat *runiflpp* function with the same number of points as the actual point pattern. The coordinates of the IRP points were directly fed to the *linearpcf* and *nndist* functions. Each image was run six times and averaged. To simplify the process, the analysis was automated using a Java script run on Eclipse. Network features and the total number of nucleoids were directly extracted from the Mitomate tracker analysis, except for the connectivity that was defined as the total number of junctions (J)/total number of mitochondrial ends (E) for each individual cell^19^.

To compare the effect of nucleoid and mitochondrial features on the pcf with random distributions, we generated random point patterns with a similar density as that of the actual point patterns (distinct from the IRP used above to correct for network effects but still fed directly to spatstat) using the same Java script. However, as each of these IRPs represent a specific distribution that can somewhat vary from the expected random distribution (especially when point density is low), 6 distributions were averaged for each experiment to avoid measuring effects due to specific random distributions. In the case of experiments where nucleoids were randomly removed, an individual distribution with points removed was considered as the actual data that was normalised over the average of 6 random distributions.

### Analysis of spatial distribution

8-bit nucleoid images and segmented mitochondria networks were manually separated into perinuclear and peripheral mitochondrial clusters using ImageJ. The perinuclear region was defined based on the relative distribution of nucleoid from the nucleus. The perinuclear area corresponds to 1/3rd of the total cellular area (long axis distance from nuclear membrane ~ 23 µm). However, care was taken to keep individual mitochondrial clusters intact when separating the mitochondrial network (each independent cluster were labeled as either perinuclear or peripheral). These images were then used to measure nucleoid size (ImageJ—*Analyse Particle* function).

### Data analysis and statistics

All data analysis was done in R. To quantify the entropy, pcf values for each distance first needed to be converted into a character string. This was achieved by first converting the decimal numbers into a whole number between 1 and 26 and attributing a letter to each number. The entropy of the resulting character string was then calculated using the *entropy* function of the R package acss^35^. The PCA was done using the R function *prcomp* while Pearson correlations were determined using the *ggcorr* function (GGally package).

Statistical analysis was done using Student’s t test (between 2 groups) or one-way ANOVA with a tukey post hoc test (multiple comparisons). Differences between nucleoid distributions were calculated using a KS test (*ks.test* function from the stats package). Linear regressions for correlation analysis were calculated using a lm method with y ~ log(x) as the general formula.

## Supplementary Information

Supplementary Figures.

## Data Availability

The datasets generated during the current study are available from the corresponding author on reasonable request.

## Code availability

The code for Mitomate tracker is available on Github (https://github.com/GermainLab/mitomate).

## References

[1] Sasaki T, Sato Y, Higashiyama T, Sasaki N (2017). Live imaging reveals the dynamics and regulation of mitochondrial nucleoids during the cell cycle in Fucci2-HeLa cells. Sci. Rep..

[2] Giacomello M, Pyakurel A, Glytsou C, Scorrano L (2020). The cell biology of mitochondrial membrane dynamics. Nat. Rev. Mol. Cell Biol..

[3] Barsh GS (2019). Mitochondrial fusion is required for regulation of mitochondrial DNA replication. PLOS Genet..

[4] El-Hattab, A. W., Craigen, W. J., Wong, L. J. C. & Scaglia, F. in *GeneReviews((R))* (eds. Adam, M. P. *et al.*) (1993).

[5] Viscomi C, Zeviani M (2017). MtDNA-maintenance defects: Syndromes and genes. J. Inherit. Metab. Dis..

[6] Friedman JR (2011). ER tubules mark sites of mitochondrial division. Science.

[7] Pagliuso A, Cossart P, Stavru F (2018). The ever-growing complexity of the mitochondrial fission machinery. Cell. Mol. Life Sci. (CMLS).

[8] Lewis SC, Uchiyama LF, Nunnari J (2016). ER-mitochondria contacts couple mtDNA synthesis with mitochondrial division in human cells. Science.

[9] Ban-Ishihara R, Ishihara T, Sasaki N, Mihara K, Ishihara N (2013). Dynamics of nucleoid structure regulated by mitochondrial fission contributes to cristae reformation and release of cytochrome c. Proc. Natl. Acad. Sci. U.S.A..

[10] Almutawa W (2019). The R941L mutation in MYH14 disrupts mitochondrial fission and associates with peripheral neuropathy. EBioMedicine.

[11] Parone PA (2008). Preventing mitochondrial fission impairs mitochondrial function and leads to loss of mitochondrial DNA. PLoS ONE.

[12] Ota A, Ishihara T, Ishihara N (2020). Mitochondrial nucleoid morphology and respiratory function are altered in Drp1-deficient HeLa cells. J. Biochem..

[13] Ishihara T (2015). Dynamics of mitochondrial DNA nucleoids regulated by mitochondrial fission is essential for maintenance of homogeneously active mitochondria during neonatal heart development. Mol. Cell. Biol..

[14] Kukat C (2011). Super-resolution microscopy reveals that mammalian mitochondrial nucleoids have a uniform size and frequently contain a single copy of mtDNA. Proc. Natl. Acad. Sci. U.S.A..

[15] Tauber J (2013). Distribution of mitochondrial nucleoids upon mitochondrial network fragmentation and network reintegration in HEPG2 cells. Int. J. Biochem. Cell Biol..

[16] Osman C, Noriega TR, Okreglak V, Fung JC, Walter P (2015). Integrity of the yeast mitochondrial genome, but not its distribution and inheritance, relies on mitochondrial fission and fusion. Proc. Natl. Acad. Sci. U.S.A..

[17] Jajoo R (2016). Accurate concentration control of mitochondria and nucleoids. Science.

[18] Iborra FJ, Kimura H, Cook PR (2004). The functional organization of mitochondrial genomes in human cells. BMC Biol..

[19] Ouellet M, Guillebaud G, Gervais V, Lupien St-Pierre D, Germain M (2017). A novel algorithm identifies stress-induced alterations in mitochondrial connectivity and inner membrane structure from confocal images. PLOS Comput. Biol..

[20] Tinevez JY (2017). TrackMate: An open and extensible platform for single-particle tracking. Methods.

[21] Baddeley A, Rubek E, Turner R (2015). Spatial Point Patterns: Methodology and Applications with R.

[22] Shannon CE (1948). A mathematical theory of communication. Bell Syst. Tech. J..

[23] Shannon CE (1948). A mathematical theory of communication. Bell Syst. Tech. J..

[24] Vanstone JR (2016). DNM1L-related mitochondrial fission defect presenting as refractory epilepsy. Eur. J. Hum. Genet. (EJHG).

[25] Silva Ramos E (2019). Mitochondrial fusion is required for regulation of mitochondrial DNA replication. PLoS Genet.

[26] Kamerkar SC, Kraus F, Sharpe AJ, Pucadyil TJ, Ryan MT (2018). Dynamin-related protein 1 has membrane constricting and severing abilities sufficient for mitochondrial and peroxisomal fission. Nat. Commun..

[27] Smirnova E, Shurland D-L, Ryazantsev SN, van der Bliek AM (1998). A human dynamin-related protein controls the distribution of mitochondria. J. Cell Biol..

[28] Korobova F, Gauvin TJ, Higgs HN (2014). A role for myosin II in mammalian mitochondrial fission. Curr. Biol..

[29] Sharma G (2021). Characterization of a novel variant in the HR1 domain of MFN2 in a patient with ataxia, optic atrophy and sensorineural hearing loss. bioRxiv..

[30] Kim BJ (2017). Discovery of MYH14 as an important and unique deafness gene causing prelingually severe autosomal dominant nonsyndromic hearing loss. J. Gene Med..

[31] Wang M (2020). A novel MYH14 mutation in a Chinese family with autosomal dominant nonsyndromic hearing loss. BMC Med. Genet..

[32] Donaudy F (2004). Nonmuscle myosin heavy-chain gene MYH14 is expressed in cochlea and mutated in patients affected by autosomal dominant hearing impairment (DFNA4). Am. J. Hum. Genet..

[33] Vandeleur D (2019). Novel and lethal case of cardiac involvement in DNM1L mitochondrial encephalopathy. Am. J. Med. Genet. Part A.

[34] Martens K (2020). Case Report: Calpainopathy presenting after bone marrow transplantation, with studies of donor genetic content in various tissue types. Front. Neurol..

[35] Gauvrit, H. S., Soler-Toscano, F. & Zenil, H. Algorithmic complexity for psychology. A user-friendly implementation of the coding theorem method. arXiv 1409.4080 (2014). 10.3758/s13428-015-0574-3 25761393

