# Supplementary material for: A new automated tool to quantify nucleoid distribution within mitochondrial networks
Source: Sci Rep. 2021 Nov 23;11:22755. doi: 10.1038/s41598-021-01987-9 (PMC8610998; doi:10.1038/s41598-021-01987-9)
Supplement: Supplementary file 1 — Supplementary Figures. [file 41598_2021_1987_MOESM1_ESM.pdf]

Supplementary information

A new automated tool to quantify nucleoid distribution within mitochondrial networks

Hema Saranya Ilamathi, Mathieu Ouellet, Rasha Sabouny, Justine Desrochers-Goyette, Matthew A. Lines, Gerald Pfeffer, Timothy E. Shutt, Marc Germain

This file contains:
Supplementary Figures (1-2)

Figure S1

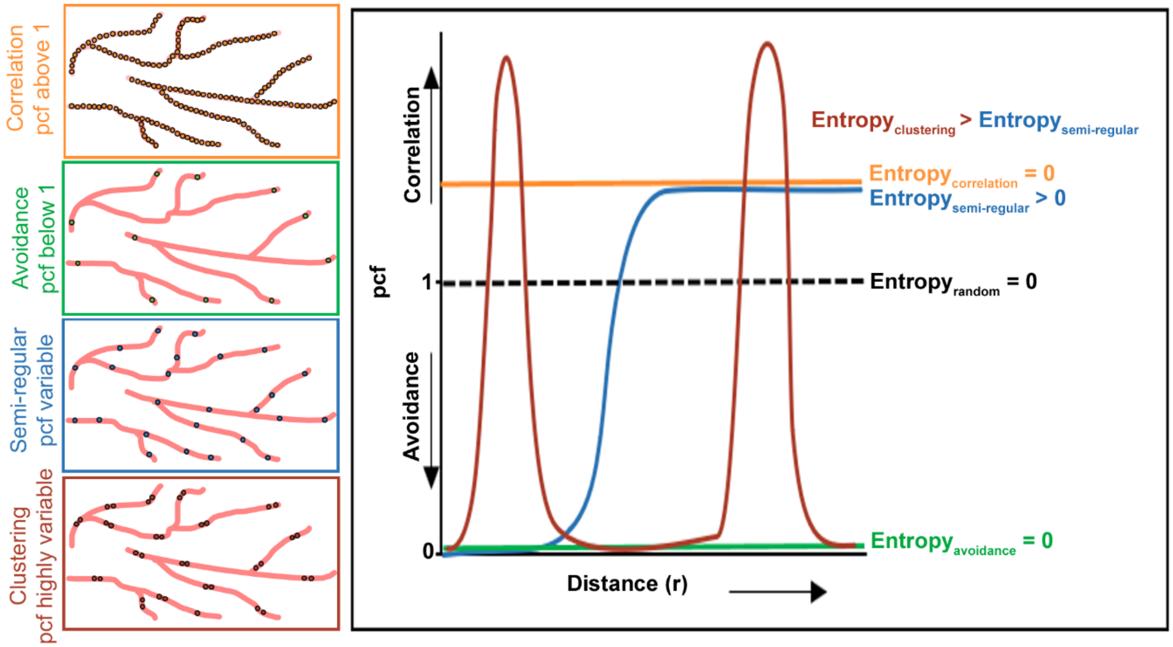

Supplementary figure 1: Entropy, a measure of variability in nucleoid distribution. The left panel represent different possible nucleoid distributions within mitochondrial networks. The right panel shows the expected pcf curves for the same distributions. Pcf value above 1 suggest correlation and anything below 1 suggest avoidance between nucleoids. The entropy, calculated from the pcf curves, increases with increasing variability of the pcf curve (an horizontal line has an entropy value of zero).

Figure S2

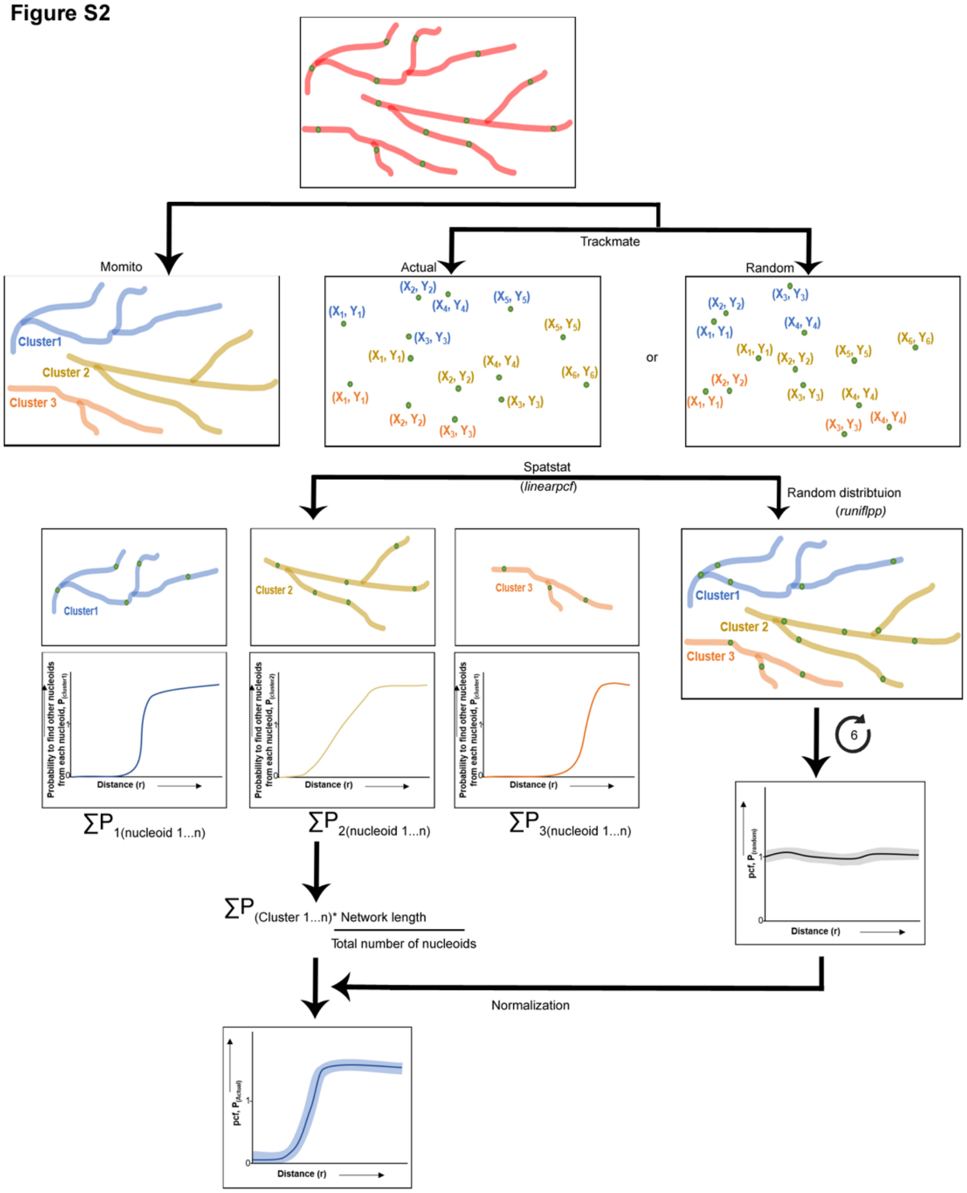

Supplementary figure 2: Workflow of Mitomate tracker. The preprocessed mitochondrial images are analyzed by Momito to extract mitochondrial network features. The coordinates of nucleoid in actual or random distributions are extracted by the Image J plugin Trackmate. Based on this information, Spatstat measures the probability of nucleoid distribution in each individual mitochondrial cluster (independent mitochondrial network). Overall nucleoid distribution is calculated by summing up the probability in all mitochondrial clusters. To take into account the effect of network features and nucleoid density, 6 random distributions (using runiflpp function) are averaged and used to normalize the actual distribution.
